# Composition-Property Relationships of pH-Responsive Poly[(2-vinylpyridine)-co-(butyl methacrylate)] Copolymers for Reverse Enteric Coatings

**DOI:** 10.3390/pharmaceutics15020454

**Published:** 2023-01-30

**Authors:** Kyle Brewer, Anton Blencowe

**Affiliations:** Applied Chemistry and Translational Biomaterials (ACTB) Group, Centre for Pharmaceutical Innovation (CPI), UniSA Clinical and Health Sciences, University of South Australia, Adelaide, SA 5000, Australia

**Keywords:** reverse enteric coating, pH-responsive polymer, taste-masked, drug delivery, spray coating

## Abstract

The taste-masking of bitter-tasting active pharmaceutical ingredients is key to ensuring patient compliance when producing oral pharmaceutical formulations. This is generally achieved via the incorporation of pH-responsive, reverse enteric polymers, that prevent the dissolution of the formulation in the oral environment, but rapidly mediate it within the gastric environment. Reverse enteric polymers are commonly applied as coatings on oral dosage forms via spray atomisation (e.g., fluidised-bed spray coating), and generally exhibit the most efficient taste-masking. However, currently used reverse enteric coatings require high mass gains (% *w*/*w*) during coating to mediate taste-masking, and thereby exhibit delayed release within the gastric environment. Therefore, there remains a need for the development of new reverse enteric coatings, that can efficiently taste-mask at low mass gains and maintain rapid release characteristics within the gastric environment. Herein we report the synthesis and evaluation of a series of addition copolymers of 2-vinylpyridine and butyl methacrylate, methyl methacrylate and isobornyl methacrylate. The thermal, solubility, and water absorption properties of the copolymers were effectively tuned by altering the mol% fraction of the constitutive monomers. Based on their physical properties, selected copolymers were preliminarily evaluated for their compatibility with fluidised-bed spray coating, and effectiveness as taste-masking reverse enteric coatings. The copolymers poly[(2-vinylpyridine)-co-(butyl methacrylate)] (mol% ratio 40:60) and poly[(2-vinylpyridine)-co-(butyl methacrylate)-co-(methyl methacrylate)] (mol% ratio 40:50:10) were found to exhibit excellent taste-masking properties following fluidised-bed spray coating onto Suglets^®^ sugar spheres. Suglets^®^ bearing a film coating of either copolymer (5.2–6.5% *w*/*w* mass gain) were found to effectively impede the release of a model drug formulation for up to 72 h in a simulated salivary environment, and rapidly release it (<10 min) within a simulated gastric environment. The results demonstrated the potential of poly[(2-vinylpyridine)-co-(butyl methacrylate)] copolymers to form effectively taste-masked, reverse enteric dosage forms, and suggested that these copolymers may provide improved performance compared to currently available polymers.

## 1. Introduction

One of the most common applications of pH-responsive polymers, is in the formation of protective coatings of pharmaceutical formulations for oral delivery. It is desirable for pharmaceutical formulations to be delivered through oral administration via the stomach or intestines, and therefore, formulations are often designed to be highly soluble once the target environment is reached [1]. For example, if the active drug is acid labile and loses functionality when exposed to the low-pH gastric environment (pH 1.5–3.5) a pH-responsive coating can be used to prevent the release of the formulation until the higher pH of the intestines (pH 5–6) is reached [2]. These types of intestine-targeting polymer coatings are termed ‘enteric’ and commonly incorporate polyvinyl acetate and methacrylic acid-ethyl acrylate copolymers [1,2,3]. In contrast, when drug dissolution within the stomach is desired, ‘reverse-enteric’ coatings are employed. Reverse-enteric coatings prevent dissolution within the higher pH oral environment (pH 5–7), but dissolve rapidly once the low-pH gastric environment is reached [1]. Generally, reverse-enteric coatings are used for taste-masking to avoid patient non-compliance due to an unpalatable pharmaceutical formulation [2], and employ tertiary amine-based acrylate copolymers. These polymers are commonly synthesised via conventional free-radical polymerisation (CFRP) [4]. 

Prominent examples of commercially available polymers used for reverse enteric coatings include the Eudragit^®^ family (Eudragit^®^ E 100, E12.5, and E PO). Reviewed elsewhere [5], these polymers are commonly used for immediate-release formulations that require taste-masking. Available in a range of forms, including granules (E 100), organic solutions (E 12.5), and powders (E PO), these Eudragit^®^ polymers are soluble in the gastric environment (and at pH values < 5) and insoluble at elevated pH (pH values > 5) [5]. Reverse enteric Eudragit polymers have been used in the production of a range of taste masked dosage forms, such as microparticles [6], beads [7], capsules [8], and 3D printed [9] or pan coated [10] tablets. However, the complete containment, and effective taste-masking of bitter-tasting active pharmaceutical ingredients (APIs) within the oral cavity remains challenging to mediate. Unique challenges are posed by the properties of reverse enteric copolymers (e.g., the swelling and permeability characteristics of Eudragit^®^ copolymers), and the required dosage form. 

For dosage forms that do not prevent contact between the bitter API and the oral cavity (pH 6.8–7.4) [11], such as microbeads (or microparticles) composed of a spherical matrix containing a mixture of the reverse enteric polymer and API, the encapsulated API is often prematurely released within the oral cavity [12]. As a result, the bitter taste of the API is only suppressed when relatively large amounts of the constitutive reverse enteric polymer are used, which limits the capacity for drug loading. For example, microbeads containing propranolol hydrochloride were prepared with Eudragit^®^ E PO via prilling, to produce a taste-masked paediatric formulation for oral administration [7]. The greatest taste-masking was achieved with microbeads exhibiting a 1:25 ratio of propranolol:matrix (equivalent to ~96% *w*/*w* matrix). While the formulation limited the release of propranolol to 3.3% following 2.5 min at pH 6.8, this could only be achieved at a low drug loading of ~38 mg·g^−1^ (mg of propranolol per g of microbead, equivalent to ~3.8% *w*/*w* propranolol) [7]. Low drug loadings may be problematic for APIs that require high doses or dosage forms with size considerations, and the use of large amounts of reverse enteric polymer may raise concerns for patient safety [6]. 

Dosage forms that provide a barrier between the bitter API and the oral cavity, such as microcapsules (also called micropellets) bearing reverse enteric film coatings, can exhibit more efficient taste-masking properties. For example, microcapsules containing the antiprotozoal drug ornidazole were film coated via fluidised-bed spray coating, with the reverse enteric polymer Kollicoat^®^ Smartseal 30D, at a 40% *w*/*w* mass gain [13]. The microcapsules limited release of ornidazole to ≤5% following 5 min at pH 6.8, and only reached <15% after 120 min. Comparatively, similar performance to the Eudragit^®^ E PO microbeads was achieved with significantly less of the Kollicoat^®^ material. Further, when a similarly high ratio of Kollicoat^®^ to drug was used (80% *w*/*w*, cf., 96% *w*/*w*), superior taste-masking performance was achieved, as no ornidazole was released in 120 min. Whilst microcapsules are advantageous for improved taste-masking performance, microbeads can exhibit more rapid drug release within the gastric environment. In the previous examples, the Eudragit^®^ microbeads achieved 100% drug release within 5 min (pH 1.2) [7], whereas the Kollicoat^®^ microcapsules achieved ~90% release within 45 min (pH 1.0) [13]. In many cases, the selection of a commercially available reverse enteric polymer and the dosage form it is incorporated into, requires a trade-off between taste-masking performance, and rapid release within the gastric environment.

The limited drug loading and release of APIs under simulated oral cavity conditions for certain formulations highlights the need for reverse enteric coatings that effectively contain bitter APIs and mediate rapid release within the gastric environment. However, in the development of new reverse enteric coatings, their intended application method is an important consideration. Film coating via spray atomisation is one of the most common and facile methods for coating pharmaceutical formulations [14]. This process involves dissolving the polymer in a solvent (organic or aqueous) before atomising the solution using compressed air. The droplets formed during atomisation deposit and spread across the substrate, and as the solvent evaporates a film is formed. Although various types of equipment can be used, processing utilising a fluidised-bed is favourable as it affords high drying efficiency and is compatible with coating formulations dispersed or solubilised in either aqueous or organic solvents [1,15]. Furthermore, the diminutive size of ‘benchtop’ fluidised-bed instruments makes them highly suited to laboratory-scale prototyping, as small batches (~500 g) can be readily produced [16].

In the current work, we report the synthesis and characterisation of a series of novel pH-responsive copolymers and evaluate their application as reverse-enteric coatings via fluidised-bed spray coating. 2-Vinylpyridine (VP) was selected as the pH-responsive moiety through which to develop an appropriately pH-responsive polymer. Given the reported *pK_a_* value of poly(2-vinylpyridine) (PVP) homopolymers (~4.5 [17]), it was hypothesised that via addition polymerisation with a methacrylate monomer(s), a pH-responsive copolymer with suitable pH-responsiveness and compatibility with fluidised-bed spray coating, could be synthesised. Due to the differences in *T_g_* values exhibited by homopolymers of VP compared to butyl methacrylate (BMA) (PVP *T_g_* = 104 °C [18], PBMA *T_g_* = 20–55 °C [18]), incorporation of different ratios of VP and BMA in a random copolymer, may enable tuning of the *T_g_* values of resultant copolymers to an appropriate processing temperature, whilst maintaining pH-responsivity. For example, when fluidised-bed spray coating an aqueous dispersion, the minimum film-formation temperature (MFF) is impacted by the *T_g_* value of the constitutive pH-responsive copolymer [19]. An appropriate *T_g_* value is required to ensure the MFF temperature is below the spray coating temperature, to facilitate coalescence of the polymer particles and cohesive film formation. Two additional, high-*T_g_* (cf., BMA) monomers—methyl methacrylate (MMA) and isobornyl methacrylate (IBMA)—were selected as additional target monomers to make a range of *T_g_* values accessible, as homopolymers of MMA or IBMA generally exhibit higher *T_g_* values than homopolymers of BMA or VP (i.e., PMMA *T_g_* = 38–150 °C [18] and PIBMA *T_g_* = 170–206 °C [20]). Further, the mol% fraction of VP in the copolymer structure, and incorporation of comparatively hydrophobic monomers, would also alter the hydrophilicity of the resultant copolymer and enable the tuning of water absorption and solubility of a coating as a result. 

To that end, a library of pH-responsive copolymers was synthesised with varying ratios of VP and BMA, and MMA or IBMA. The physicochemical properties of these copolymers were investigated, to determine which copolymers exhibited favourable solubility (insoluble at pH 7.4, soluble at pH < 2), low water absorption, and a suitable glass transition temperature (*T_g_*) to enable spray coating. These properties were considered necessary to afford a uniform and cohesive coating following spray coating. The physicochemical properties of the synthesised copolymers were investigated through solubility, water absorption, and thermal analysis experiments. Selected copolymers were evaluated for their compatibility with fluidised-bed spray coating and potential effectiveness as reverse enteric coatings for taste-masking.

## 2. Materials and Methods

### 2.1. Materials

All reagents were used as received unless otherwise specified. 2-Vinylpyridine (VP, 97%), butyl methacrylate (BMA, 99%), methyl methacrylate (MMA, 99%), and isobornyl methacrylate (IBMA, 92.5%) were purchased from Sigma-Aldrich (St. Louis, MO, USA). Inhibitors were removed from vinyl and methacrylate monomers via basic aluminium oxide before use. Azo*bis*isobutyronitrile (AIBN, 12% *w*/*w* in acetone), poly(ethylene glycol) (number average molecular weight (*M_n_*) = 10 kDa, PEG 10k), rhodamine b (RhB, ≥95%), basic aluminium oxide, lithium chloride (LiCl, technical grade) and deuterated chloroform (CDCl_3_), were purchased from Sigma-Aldrich (St. Louis, MO, USA). Analytical grade 1,4-dioxane, ethanol (EtOH, 100% undenatured), potassium chloride (KCl), hydrochloric acid (HCl, 36.5–38.0%), sodium acetate (NaOAc), and HPLC grade tetrahydrofuran (THF) and dimethylformamide (DMF) were purchased from ChemSupply (Gillman, Australia). Poly(methyl methacrylate) (PMMA) standards for gel permeation chromatography (GPC) were purchased from Polymer Standards Service GmbH (Mainz, Germany). Phosphate buffered saline (PBS) tablets were purchased from Sigma-Aldrich (St. Louis, MO, USA). Solutions for PBS were prepared per the manufacturer’s guidelines. An Arium Pro Ultrapure Water System (Sartorius, Goettingen, Germany) was used to produce high-purity water (≥18.2 MΩ·cm). pH 1.5 solutions were prepared from KCl (0.2 M) and HCl (0.2 M). Nitrogen and argon (Ar) (ultra-high purity, ≥99.999%) gases were purchased from BOC Ltd. (North Ryde, Australia). Suglets^®^ sugar spheres (12/14 mesh, 1.4–1.7 mm diameter) were provided by Colorcon^®^ (Scoresby, Victoria, Australia).

### 2.2. Instrumentation

Gas chromatography (GC) was performed on a Shimadzu GC-2010 gas chromatograph (Kyoto, Japan). Proton nuclear magnetic resonance spectroscopy (^1^H NMR) was conducted on a Bruker Avance III HD 600 MHz spectrometer (Billerica, MA, USA). GPC was performed using a Shimadzu Prominence liquid chromatography (LC) system (Kyoto, Japan), fitted with a differential refractive index detector (Shimadzu, RID-20A) and two Shimadzu columns in series (GPC-80MD and GPC-804D). Modulated differential scanning calorimetry (MDSC) and thermogravimetric analysis (TGA) were conducted using TA Instruments (New Castle, WY, USA) DSC 250 and TGA5500, respectively. Ultraviolet-visible (UV-vis) spectrophotometry was performed on an Evolution 260 Bio spectrophotometer, and temperature-controlled using a single-cell Peltier system (Thermo Scientific, Waltham, MA, USA). Fluidised-bed spray coating was conducted using a benchtop Mini Coater/Drier 2 (Caleva Process Solutions Ltd., Dorset, UK).

### 2.3. Procedures

#### 2.3.1. Synthesis of Poly[(2-vinylpyridine)_x_-co-(butyl methacrylate)_y_]

The copolymers of poly[(2-vinylpyridine)_x_-co-(butyl methacrylate)_y_] (PVB x/y) were synthesised with x:y ratios of 40:60, 50:50, and 60:40 mol% (VP:BMA). A representative description of the synthesis of PVB 50/50 is provided.

Inhibitor-free VP (25.6 mL, 238 mmol) and BMA (37.8 mL, 238 mmol) (mol% VP:BMA 50:50), and AIBN solution (6.51 mL, 4.8 mmol)) were combined ([total monomer]:[initiator] 100:1 mole ratio) in 1,4-dioxane (50% *v*/*v*) under an inert atmosphere (Ar) and heated at reflux with stirring. Additional AIBN solution (6.51 mL, 4.8 mmol) was added after 1 and 2 h to maximise monomer conversion. After 3 h, the reaction was cooled (~25 °C), the crude reaction mixture (CRM) was diluted with THF (~20% *v*/*v*), and the copolymer product precipitated from hexanes (1:10 *v*/*v* CRM:hexanes) in a conical flask. The supernatant was decanted, and the precipitate was redissolved in THF (~75 mL), transferred to a round-bottomed flask, concentrated in vacuo (40 °C, 10 mbar), and then dried in vacuo (23 °C, 0.02 mbar) to afford the polymer as a pale-yellow solid. The percentage of monomer conversion (determined by GC) was 95% and the isolated yield was >99%. GPC *M_n_* = 16.2 kDa, *M_w_* = 31.9 kDa, *Ð* = 1.97. ^1^H NMR (600 MHz, 25 °C, CDCl_3_) δ_H_ 8.45 (br s, CH, VP repeat unit (RU)), 7.44 (br s, CH, VP RU), 6.99–6.50 (m, CH, VP RU), 3.87 (br s, OCH_2_, BMA RU), 3.53–2.32 (m, CH, VP RU), 2.32–1.02 (m, CH_2_, BMA and VP RUs), 1.02–0.16 (m, CH_3_, BMA RU) ppm. GPC chromatograms and ^1^H NMR spectra of the PVB copolymers are provided in the Supplementary Materials, Figures S1 and S2, respectively.

#### 2.3.2. Synthesis of Poly[(2-vinylpyridine)_x_-co-(butyl methacrylate)_y_-co-(methyl methacrylate)_z_]

The copolymers of poly[(2-vinylpyridine)_x_-*co*-(butyl methacrylate)_y_-*co*-(methyl methacrylate)_z_] (PVBM x/y/z) were synthesised with x:y:z ratios of 40:50:10, 30:50:20, 30:40:30, and 40:40:20 mol% VP:BMA:MMA. A representative description of the synthesis of PVBM 40:50:10 is provided.

Inhibitor-free VP (20.5 mL, 190 mmol), BMA (37.8 mL, 238 mmol) and MMA (5.07 mL, 47 mmol) (mol% VP:BMA:MMA 40:50:10), and AIBN solution (6.51 mL, 4.8 mmol)) were combined ([total monomer]:[initiator] 100:1 mole ratio) in 1,4-dioxane (50% *v*/*v*) under an inert atmosphere (Ar) and heated at reflux with stirring. Additional AIBN solution (6.51 mL, 4.8 mmol) was added after 1 and 2 h to maximise monomer conversion. After 3 h, the reaction was cooled (~25 °C), the CRM was diluted with THF (~20% *v*/*v*), transferred to a round-bottomed flask, and the copolymer product was obtained by drying the CRM in vacuo (10 mbar, 80 °C). The copolymer product was obtained as a pale-yellow solid. The percentage monomer conversion (determined by GC) was 99% and the isolated yield was >98%. GPC *M_n_* = 14.8 kDa, *M_w_* = 32.4 kDa, *Ð* = 2.19. ^1^H NMR (600 MHz, 25 °C, CDCl_3_) δ_H_ 8.45 (br s, CH, VP repeat unit (RU)), 7.41 (br s, CH, VP RU), 6.97–6.50 (m, CH, VP RU), 3.90 (br s, OCH_2_, BMA RU), 3.54 (br s, OCH_3_, MMA RU), 3.25–2.07 (m, CH, VP RU), 2.07–1.01 (m, CH_2_, VP, BMA and MMA RU), 1.01–0.13 (m, CH_3_, BMA RU) ppm. GPC chromatograms and ^1^H NMR spectra of the PVBM copolymers are provided in the Supplementary Materials, Figures S1 and S3, respectively.

#### 2.3.3. Synthesis of Poly[(2-vinyl pyridine)_x_-co-(butyl methacrylate)_y_-co-(isobornyl methacrylate)_z_]

The copolymers of poly[(2-vinylpyridine)_x_-*co*-(butyl methacrylate)_y_-*co*-(isobornyl methacrylate)_z_] (PVBI x/y/z) were synthesised with x:y:z ratios of 50:45:5, 50:40:10, 50:35:15, 50:30:20, and 40:40:20 mol% VP:BMA:IBMA. A representative synthesis of the PVBI 50:30:20 copolymer is given below.

Inhibitor free VP (20.50 mL, 190 mmol), BMA (18.00 mL, 113 mmol), and IBMA (17.25 mL, 76. mmol), and AIBN solution (5.25 mL, 3.1 mmol) were combined ([total monomer]:[initiator] 122:1 mole ratio) in 1,4-dioxane (50% *v*/*v*) under an inert atmosphere (Ar) and heated at reflux with stirring. AIBN (5.25 mL, 3.1 mmol) was added again after 1 and 2 h to maximize monomer conversion. After 3 h, the reaction was cooled (~25 °C), and the solvent was removed in vacuo. Further drying was conducted at 80 °C in vacuo (0.01 mbar) to afford the polymer as a pale-yellow solid. The percentage conversion (determined by GC) was >99%, and the isolated yield was >98%. GPC *M*_n_ = 17.7 kDa, *M*_w_ = 34.0 kDa, *Đ* = 1.92. ^1^H NMR (600 MHz, 25 °C, CDCl_3_): *δ*_H_ 8.40 (br s, CH, VP repeat unit (RU)), 7.39 (br s, CH, VP RU), 7.10–6.20 (m, CH, VP RU), 4.45−3.05 (m, OCH_2_, BMA RU; OCH, IBMA RU), 3.00–2.05 (m, CH, VP RU), 1.97−1.0 (m, CH_2_, VP, BMA and IBMA RU; CH, IBMA RU), 0.97–0.45 (m, CH_3_, BMA and IBMA RU) ppm. GPC chromatograms and ^1^H NMR spectra of the PVBI copolymers are provided in the Supplementary Materials, Figures S1 and S4, respectively.

#### 2.3.4. Gas Chromatography

Syntheses were monitored by analysing 50 µL aliquots of the CRM diluted in DCM (1.5 mL) at predetermined time points. Monomer consumption was determined from the relative decrease in peak area compared to their pre-polymerisation (t_0_) concentrations, using 1,4-dioxane as an internal reference. Fitted with a flame ionisation detector (FID) and a Supelco SPB-35 column (30.0 m × 0.25 mm, 0.25 µm) (Bellefonte, PA, USA) and using nitrogen carrier gas, the GC was operated in linear velocity mode. A linear velocity of 56.5 cm·s^−1^ was used, with the sample injection port and FID temperature both at 250 °C. For all samples, a split injection (split ratio 50:1) and injection volume of 1.0 µL were used, using a temperature program consisting of 40 °C (1 min hold) to 180 °C (2 min hold) at 22.50 °C·min^−1^. 

#### 2.3.5. Gel Permeation Chromatography

Synthesised copolymers were analysed for their molecular weight characteristics via GPC. The Shimadzu LC system was operated at a column oven temperature of 40 °C, and a flow rate of 1 mL·min^−1^, using THF as the eluent. Prior to injection at an injection volume of 50 µL, copolymer samples (5 mg·mL^−1^) were filtered using 0.45 µm nylon syringe filters. A conventional column calibration of narrow molecular weight PMMA standards was used to determine the molecular weight characteristics of the copolymers, via the Shimadzu LabSolutions software (v5.93). The above parameters were used to determine the solubilities of the synthesised copolymers, except with a column oven temperature of 70 °C and an eluent consisting of 0.1 M LiCl in DMF.

#### 2.3.6. Modulated Differential Scanning Calorimetry

MDSC analyses were conducted on powdered copolymer samples (~5–10 mg) in standard aluminium pans, under nitrogen flow (cell purge flow rate, 50 mL·min^−1^). Samples were heated from −80 to 250 °C at a ramp rate of 3 °C·min^−1^, using a modulation period of 40 s, and a modulation temperature amplitude of ±64 °C. The Universal Analysis software (v4.5A) (TA Instruments, New Castle, WY, USA) was used to determine the *T_g_* values of each copolymer, from the point of inflection between the onset and end temperatures of the transition, in the reversing heat flow (W·g^−1^) curves.

#### 2.3.7. Thermogravimetric Analysis

TGA analyses were conducted on powdered copolymer samples (~10 mg) in platinum pans, under nitrogen flow (sample purge flow rate, 25 mL·min^−1^). Samples were heated from 30 to 600 °C, at a ramp rate of 20 °C·min^−1^. The onset values for the thermal degradation of each copolymer were determined from the derivative weight (%·°C^−1^) curves. Weight loss values were determined from the step change of the major degradation event observed in the weight (%) curves. Both the onset and weight loss values were determined using TA Instruments Universal Analysis software (v4.5A).

#### 2.3.8. Copolymer Water Absorption

A 15-ton hydraulic press was used to prepare powdered copolymer samples (~150 mg) as discs (13 mm diameter × 1.2 mm height). The copolymer discs were dried for 72 h in vacuo (0.01 mbar, 23 °C), then weighed and submerged in PBS (20 mL, pH 7.4) in sealed vials and stored at 37 °C. The copolymer discs were removed periodically from the solution and any solution present on the disc surface was removed using lint-free tissue paper (Kimwipes™) prior to weighing. The mass of the absorbed PBS solution was then calculated from the increase in disc mass. The copolymer discs were monitored for 40–60 d. All copolymers were analysed in triplicate, and water absorption values were reported as the mean ± std. dev.

#### 2.3.9. Copolymer Solubility

Powdered copolymer samples (~10 mg) were combined with 1 mL of a pH 1.0, 1.5, or 2.0 solution, in microcentrifuge tubes. The tubes were rotary mixed for 48 h at 37 °C, then centrifuged (15 krpm, 3 min), to separate the solubilised copolymer in the supernatant solution, and the insoluble copolymer solid. A 50 μL aliquot of the supernatant was removed, diluted (1:10) in GPC mobile phase (0.1 M LiCl in DMF) and analysed via GPC. The peak area of the copolymer was correlated to a calibration curve (vide infra), to determine the concentration of the copolymer in the sample, and therefore, its solubility. 

Seven-point calibration curves were prepared for each copolymer by correlating the known mass of a copolymer standard to its peak area value obtained via GPC analysis (Supplementary Materials, Figure S5). Copolymer standards were prepared by vortex mixing (2 krpm) a 10 mg sample in a pH 1.0 solution (1 mL, 10 mg·mL^−1^) until homogeneity, and conducting a 1:10 dilution in the mobile phase. Each copolymer standard was analysed via GPC at injection volumes from 10–100 µL, to afford a seven-point calibration curve. 

#### 2.3.10. Ultraviolet-Visible Spectrophotometry

UV-vis spectrophotometry was conducted at 37 °C using a quartz cuvette (*l* = 10 mm) and stirred at the maximum stirring rate during analysis.

#### 2.3.11. Fluidised-Bed Spray Coating

A model drug formulation was prepared by solubilising PEG 10 kDa (7.5 g) and RhB (1.3 g) in 70:30% *v*/*v* EtOH:water (170 mL). The model drug formulation was introduced to the spray coater via a syringe pump operating at a flow rate of 15.2 mL·h^−1^ and atomised using an atomising air pressure of 5 PSI. Suglets^®^ (*n* = 1800) were fluidised with a fan speed of 90% and fluidisation air temperature of 40 °C, throughout the coating. The formulation was applied in a single coating cycle (45 min), after which the coated Suglets^®^ were dried in vacuo (0.02 mbar, 23 °C, 16 h), and stored in a desiccator prior to use.

Reverse enteric copolymer coating formulations were prepared by solubilising PVB 40/60, PVB 60/40, PVBM 40/50/10, or PVBM 30/50/20 in EtOH (50 mL). Reverse enteric copolymer coatings were applied using the same parameters as the model drug formulation, except the fluidisation air temperature was set to ambient temperature (23 °C). Suglets^®^ (*n* = 340) were coated in two cycles (45 min each, 90 min total) for each copolymer, to afford weight gains of 4.9–6.5% *w*/*w*. The coated Suglets^®^ were dried in vacuo after the first cycle (0.02 mbar, 23 °C, 20 min), and again after the second (16 h), then stored in a desiccator prior to use.

#### 2.3.12. Taste-Masked Formulation Stability

A simulated salivary fluid (phosphate-buffered, pH 6.8 solution) was prepared from a pH 7.4 PBS solution, via the addition of 0.1 M HCl. Suglets^®^ coated with both the model drug formulation and a reverse enteric copolymer coating were submerged in a pH 6.8 solution (3.0 mL) and stored at 37 °C, to mimic human salivary conditions. Samples were monitored periodically, and taste-masking failure was determined qualitatively, by the presence of RhB in the receiving solution.

#### 2.3.13. Taste-Masked Formulation Release

Suglets^®^ coated with both the model drug formulation and a reverse enteric copolymer coating were suspended in a pH 1.5 solution (3.5 mL) at 37 °C, under stirring, to mimic the human gastric environment. The presence of RhB in the receiving solution was monitored inline by UV-vis spectrophotometry (λ_max_ = 556 nm), to determine the initial and complete release times of RhB.

## 3. Results and Discussion

### 3.1. Synthesis and Characterisation

A series of addition copolymers of VP and BMA, incorporating MMA or IBMA, were synthesised via conventional free radical polymerisation (CFRP), with varying ratios of the monomers (Table 1). Polymerisation was initiated by AIBN, and monomer conversion was monitored via gas chromatography (GC). Additional volumes of AIBN were added hourly over 3 h, until monomer conversion exceeded 95%. The copolymer product was either purified via precipitation from hexanes or obtained after the removal of residual monomer and solvent in vacuo. In all cases, the polymers were obtained as pale-yellow solids. 

Monomer conversion and the relative mol% fraction of repeat units of the incorporated monomers were determined via GC with respect to their pre-polymerisation (t_0_) concentrations, using 1,4-dioxane as an internal reference. In all cases the targeted mol% ratios were closely achieved using this approach (Table 1), and the copolymers were designated as PVB x/y, PVBM x/y/z, or PVBI x/y/z, where x, y, and z refer to the mol% fraction of the respective monomer repeat units (RUs). 

**Figure 1:**
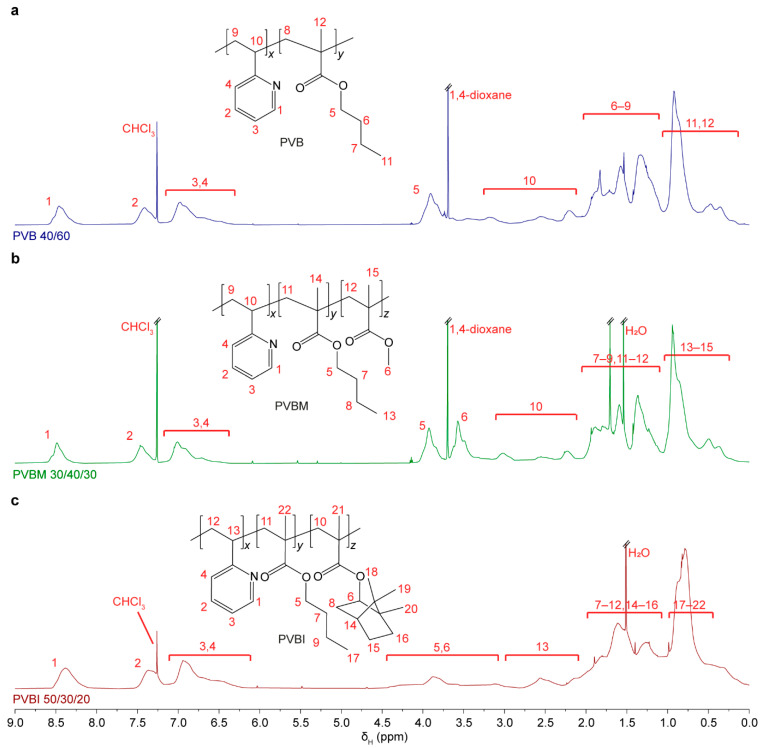
Representative ^1^H NMR spectra (600 MHz, 25 °C, CDCl_3_) of the PVB, PVBM, and PVBI series, showing (**a**) PVB 40/60, (**b**) PVBM 30/40/30, and (**c**) PVBI 50/30/20. ^1^H NMR spectra of the remaining copolymers in each series are provided in the Supplementary Materials, Figures S2–S4.

The synthesised copolymers were further characterised via ^1^H NMR spectroscopy and GPC. The broad, overlapping resonances obtained in the ^1^H NMR spectra—typical of a random copolymer—precluded accurate determination of monomer RUs to allow corroboration with the GC results (Figure 1 and Figures S2–S4). However, resonances characteristic of the VP RU aromatic protons between δ_H_ 8.40 and 6.75 ppm, and BMA RU ester methylene protons at δ_H_ 3.9 ppm, supported the successful incorporation of the two monomers in the PVB copolymers. The characteristic methoxy proton resonance of the MMA RU at δ_H_ ~3.6 ppm, confirmed the successful incorporation of MMA in the PVBM copolymers. For the PVBI copolymer series, the resonance observed at 0.75 ppm corresponded to the methyl groups of the IBMA RUs, confirming successful incorporation. The ^1^H NMR spectra also showed the presence of trace amounts of solvents used during synthesis (1,4-dioxane (δ_H_ 3.67 ppm) and THF (δ_H_ 3.75 and 1.70 ppm), and water (δ_H_ 1.52 ppm) attributed to the use of solvents that were not anhydrous. In some cases, trace amounts of unreacted monomers were observed (i.e., alkene proton resonances between δ_H_ 6.2 and 5.0 ppm). However, the presence of these impurities was considered inconsequential given their trace amounts, and the copolymers were used without further purification.

**Figure 2:**
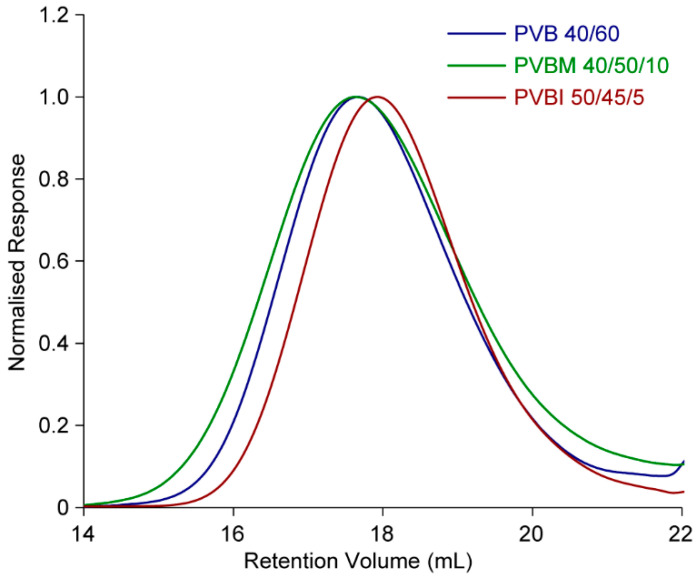
Representative normalised differential reactive index GPC chromatograms of the PVB, PVBM and PVBI copolymer series. Chromatograms of the remaining copolymers are provided in the Supplementary Materials, Figure S1.

GPC analyses of the copolymers (Figure 2) revealed similar *M_w_* values between 30–37 kDa (PVB series), 32–36 kDa (PVBM series) and 25–43 kDa (PVBI series), and broad, monomodal molecular weight distributions (*Đ* = 1.7–2.2, Table 1), consistent with dispersities that are typically observed for polymers synthesised by CFRP. It is important to note that the copolymer *M_w_* values determined from the GPC analyses (Table 1) are relative to the PMMA standards used to calibrate the system and are not absolute. Together, the results from the GC and GPC analyses, and ^1^H NMR spectra, demonstrated the successful synthesis of the copolymer series with excellent accuracy regarding the target RU mol% ratios and relatively consistent molecular weight profiles.

### 3.2. Thermal Properties

TGA and MDSC analyses were conducted on the synthesised copolymers to investigate their thermal properties, namely their degradation temperature (*T_d_*) and *T_g_*, respectively. While the *T_d_* values of the copolymers would inform the processing conditions that could be used, the *T_g_* values would affect the copolymer’s compatibility with spray coating and its performance if coated on a tablet or microparticle formulation [21,22].

**Figure 3:**
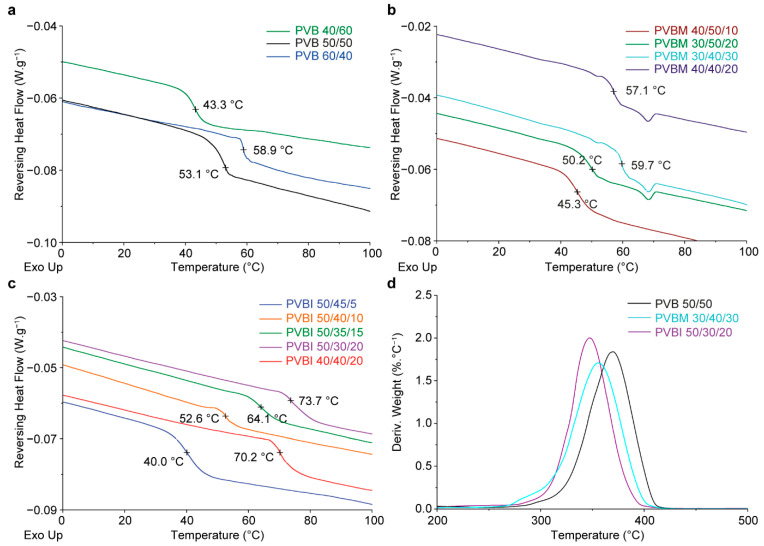
Overlays of reversing heat flow (W·g^−1^) curves obtained via MDSC for the (**a**) PVB, (**b**) PVBM, and (**c**) PVBI copolymer series, showing their respective *T_g_* values. (**d**) Derivative weight (%·°C^−1^) curves obtained via TGA for representative copolymers in the PVB, PVBM and PVBI series.

From the MDSC analyses, none of the synthesised copolymers exhibited either a defined melting (*T_m_*) or crystallisation (*T_c_*) temperature, which demonstrated that all the copolymers were amorphous. The reversing heat flow curves from the MDSC analyses of the PVB copolymers (Figure 3a) revealed a positive correlation between the mol% fraction of VP and the *T_g_* of the copolymers. PVB 60/40 had the highest mol% fraction of VP and exhibited the highest *T_g_* of 58.9 °C, whereas PVB 40/60 had the lowest *T_g_* of 43.3 °C, and PVB 50/50 had an intermediate *T_g_* of 53.1 °C (Figure 3a). This relationship results from the contribution of the VP RUs to the *T_g_* values of the resultant copolymers, as VP homopolymers generally exhibit high *T_g_* values (cf., BMA) due to the stronger intermolecular forces between VP RUs, and the bulky nature of pendant VP RUs. Whereas BMA RUs are less bulky with weaker interactions, which causes BMA homopolymers to have lower *T_g_* values.

For the PVBM copolymers, an increase in the mol% fraction of MMA by ~10% and a commensurate reduction in BMA at a constant fraction of VP (~40%) (i.e., PVBM 40/40/20 versus PVBM 40/50/10) resulted in a ~12 °C increase in *T_g_* (Figure 3b). This suggested that there was a greater contribution to the *T_g_* from MMA in the resultant copolymer (cf., BMA). Which was supported by previous reports of the dependence of the *T_g_* values of MMA copolymers, on the proportion of MMA [23,24,25,26,27]. In contrast, when the mol% fraction of VP was lowered to ~30%, and the proportion of MMA increased commensurately in PVBM 30/50/20 (cf., PVBM 40/50/10), an increase in the *T_g_* value of ~5 °C was observed. Additionally, when the proportion of MMA was increased further (and the mol% fraction of BMA reduced, in PVBM 30/50/30), a further ~10 °C increase in the *T_g_* value was observed. The relative increase in *T_g_* despite a reduction in the mol% fraction of VP, demonstrated that there was a strong positive relationship between the proportion of MMA and the *T_g_* value of the resultant copolymer [23,24,25,26,27]. This is likely due to the tendency of MMA to form syndiotactic homopolymers exhibiting higher *T_g_* values, due to the stronger interactions afforded by the relatively polar MMA RUs (cf., BMA) [18,28]. 

The *T_g_* values of the PVBI copolymers showed that a higher proportion of IBMA (e.g., PVBI 40/40/20) or VP (e.g., PVBI 50/30/20) resulted in a commensurate increase in the *T_g_* value (Figure 3c). Particularly, an increase in the IBMA content from ~5–20% (i.e., PVBI 50/45/5 cf., PVBI 50/30/20) with a constant proportion of VP (~50%) and a reduction in BMA content (from ~45–30%) resulted in a ~35 °C increase in the *T_g_* value of the resultant copolymer. The increase in the *T_g_* value likely results from the bulky and rigid pendant isobornyl groups of the IBMA RUs, which reduce chain mobility [20,29,30]. In comparison, the flexible and less bulky aliphatic chain of the BMA RUs, lowers the *T_g_* value of the resultant copolymer. The reported *T_g_* values of the homopolymers of the monomers support this reasoning [20,31]. These results supported the hypothesised ability to tune the *T_g_* values of the copolymers in each series by changing the RU ratios of the constituent monomers. Copolymers with *T_g_* values from 40–74 °C are easily accessible in these series, potentially providing compatibility with a range of processing conditions.

Representative copolymers from each series were analysed via TGA, given the high likelihood of a shared degradation mechanism amongst copolymers from the same series. The PVB 50/50, PVBM 30/40/20, and PVBI 50/30/20 copolymers exhibited similar onset values for thermal degradation (~270 °C) (Figure 3d). Followed by a single degradation event involving a weight loss of ~95–97%, which suggested a similar degradation mechanism predominated. Homopolymers of VP and BMA thermally degrade predominantly via depolymerisation, with major degradation products being monomer and low molecular weight oligomers [32,33]. When incorporated in a block copolymer of PVP and polystyrene (i.e., poly(2-vinylpyridine-*co*-styrene)), the same degradation mechanism for the homopolymer of VP is observed for the PVP block [34]. Similarly, depolymerisation is the same thermal degradation mechanism observed for both homo- and copolymers of MMA (e.g., poly[(methyl methacrylate)-*co*-(benzyl methacrylate)] [35,36]. In contrast, the major thermal degradation pathway for IBMA homopolymers is γ-H transfer from the isobornyl ring to the carbonyl group [37,38]. The single decomposition event observed for the copolymers in all three series, suggested that degradation likely occurred predominantly via depolymerisation. Importantly, whilst the temperature of the major decomposition event has little implications within the context of fluidised-bed spray coating, the favourable thermal stability of the synthesised copolymers may make them suitable for processing at elevated temperatures (i.e., up to 250 °C). For example, hot-melt extrusion has been successfully used to produce taste-masked orally disintegrating tablets, containing the reverse enteric copolymer Eudragit^®^ E PO, and ibuprofen, with improved friability and release properties compared to a commercially available dosage form [39]. Extrusion was performed at 140 °C, which is an extrusion temperature that is accessible to all of the copolymers prepared in this study.

### 3.3. Copolymer Solubility

The pH-dependent solubility of a copolymer is integral to its performance as a reverse enteric coating. Invariably, to effectively achieve stability within the oral cavity and rapid dissolution within the stomach, a significant solubility differential is required between both environments. To effectively compare the solubility of the synthesised copolymers, their solubility was evaluated in solutions simulating the gastric environment (i.e., pH 1.0, 1.5 and 2.0). The solubility of each copolymer was calculated from its concentration, as determined via GPC analysis of the aqueous solution, using relevant standard curves (Supplementary Materials, Figure S5). 

**Figure 4:**
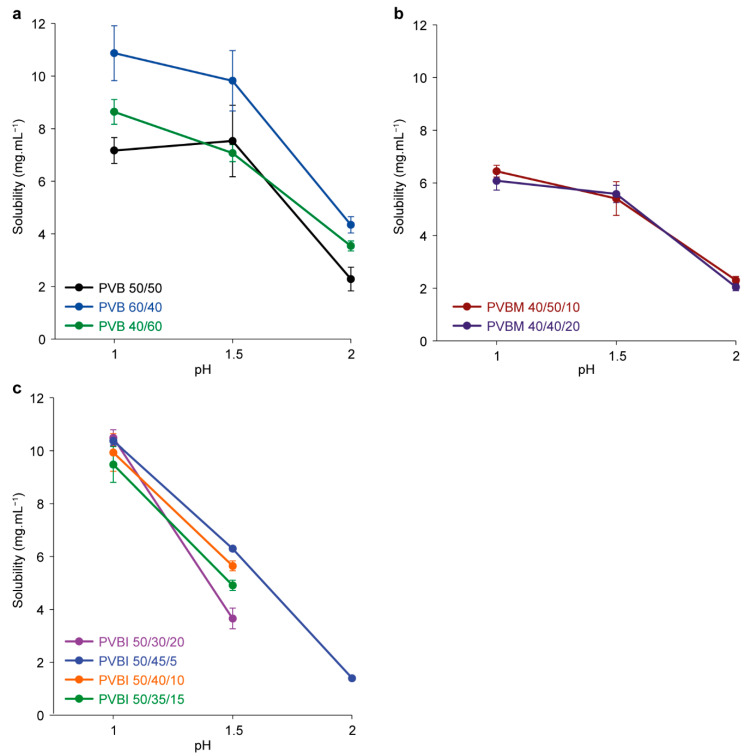
The effect of (**a**) PVB, (**b**) PVBM, and (**c**) PVBI copolymer composition on solubility at pH 1.0, 1.5, and 2.0 (37 °C). Values are reported as the mean ± std. dev. (*n* = 3). Solubility values are tabulated in the Supplementary Materials, Table S1.

The solubility of the PVB copolymers was generally positively correlated with the mol% fraction of VP (Figure 4a), and decreased with increasing pH (i.e., decreased protonation of the VP RUs). At pH 1.5, PVB 60/40 exhibited the greatest solubility (9.8 ± 1.2 mg·mL^−1^), whilst PVB 50/50 and PVB 40/60 exhibited similar solubilities (7.5 ± 1.4 and 7.1 ± 0.3 mg·mL^−1^).

In the PVBM series, PVBM 30/50/20 and 30/40/30 exhibited poor solubility (<5 mg·mL^−1^), and precluded the analysis of their solubility, as the detector response fell below the limit of quantification. PVBM 40/50/10 and PVBM 40/40/20 exhibited pH-dependent solubility (Figure 4b), with similar solubilities determined for all the test solutions (pH 1.0, 1.5 and 2.0). Overall, the solubilities of PVBM 40/50/10 and PVBM 40/40/20 at pH 1.5 (5.4 ± 0.6 and 5.6 ± 0.3 mg·mL^−1^, respectively) were lower than all of the copolymers in the PVB series. The lowered solubility of the PVBM copolymers (cf., PVB), was likely due to both the reduction in VP mol% fraction (cf., PVB 50/50), and the glassy MMA segments within the copolymer structures. The relative hydrophilicity of MMA (cf., BMA) was not sufficient to overcome this reduction in solubility. This effect was even more pronounced for PVBM 30/50/20 and 30/40/20, which exhibited even lower solubility.

Except for PVBI 40/40/20, all of the copolymers in the PVBI series were soluble at pH 1.5 (≥ ~4 mg·mL^−1^) (Figure 4c). Given that PVBI 40/40/20 contained the lowest and highest proportions of VP (40%) and IBMA (20%) respectively, it follows that it exhibited the poorest aqueous solubility due to its relatively greater hydrophobicity. As the mol% fraction of IBMA was decreased to 5%, at pH 1.5, the solubilities of the copolymers increased from 3.7 ± 0.4 mg/mL^−1^ (PVBI 50/30/20) to 6.3 ± 0.1 mg·mL^−1^ (PVBI 50/45/5), which indicated that the solubility of the copolymers was proportional to the IBMA mol% fraction, when the mol% fraction of VP was held constant at 50%. Overall, these results demonstrate that the VP mol% fraction of the copolymers mediates their solubilities, but solubility can be further tuned—in a proportional manner—through the incorporation of relatively hydrophobic monomers at a constant VP mol% fraction.

### 3.4. Water Absorption

**Figure 5:**
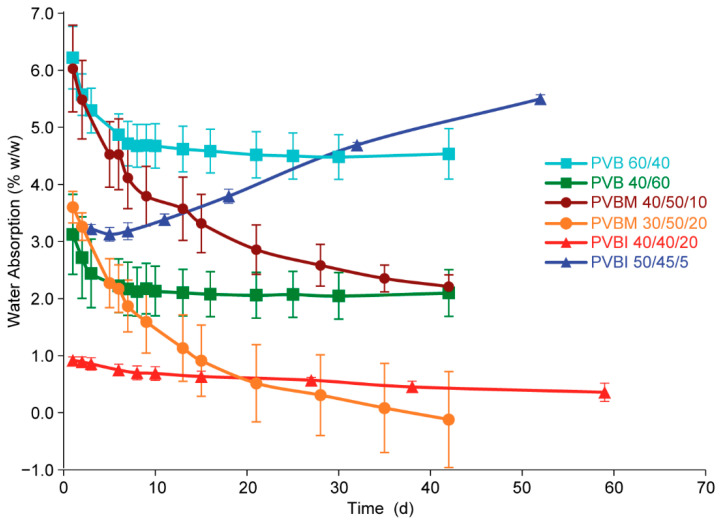
The effect of copolymer composition on the water absorption of the synthesised copolymers in PBS (pH 7.4, 37 °C). Values are reported as the mean ± std. dev. (*n* = 3). Water absorption results from the remaining copolymers are provided in the Supplementary Materials, Figure S6.

It was important to determine the water absorption properties of the copolymers, as the absorption of water could affect the integrity of the dosage form (e.g., following film coating), or the stability of an API (e.g., following permeation of water through a coating, and dissolution of the API) [40,41]. The water absorption (in % *w*/*w*) of the copolymers in each series was evaluated gravimetrically by immersing polymer discs in PBS (pH 7.4, 20 mL) at 37 °C and periodically recording their change in mass. Representative water absorption profiles of the least and most absorptive copolymers are shown in Figure 5. For all series, an initial peak in the absorption was observed over the first few days, which was attributed to the sequestration of water in the void spaces created when the polymer discs were prepared. Generally, the amount of absorbed water declined during the study period (Figure 5). For most copolymers, water absorption appeared to reach a near-constant value following ~40 d incubation (Supplementary Materials, Figure S6). 

For the PVB copolymer series, the extent of water absorption was found to be proportional to the mol% fraction of VP in the copolymer, with PVB 60/40 resulting in the greatest water absorption after reaching an apparent equilibrium between 30–40 d (~4.5% *w*/*w*), and PVB 40/60 the lowest water absorption (~2.1% *w*/*w*) within the same period (Figure 5). Such a relationship is reasonable given the relatively higher hydrophilicity of VP and is supported by the demonstrated proportional relationship between the solubility of a copolymer and its mol% fraction of VP.

Consistent with the PVB series, the water absorption exhibited by the PVBM copolymers was correlated with the mol% fraction of VP (Figure 5). The copolymer with the lowest proportion of VP, PVBM 30/50/20, showed the lowest water absorption (42 d, −0.1% ± 0.84 *w*/*w*). The apparent negative water absorption at later time points (30–40 d), was likely due to a combination of the copolymer’s low water uptake, and the limitations inherent to determining water absorption on a bulk sample gravimetrically. Regardless, when compared to PVBM 40/50/10, which had the greatest water absorption (42 d, 2.4% ± 0.2 *w*/*w*), the extent of water absorption was positively correlated with the mol% fraction of VP. When the mol% fraction of BMA was reduced in favour of a higher MMA mol% fraction (with the same VP mol% fraction) (e.g., PVBM 40/50/10 cf. 40/40/20, Supplementary Materials Figure S6) no significant difference in water absorption was observed. This suggested that water absorption was not correlated with the mol% fraction of MMA and was predominantly dependent on the mol% fraction of VP in the copolymer. 

The water absorption exhibited by the PVBI copolymers was found to be proportional to the mol% fraction of IBMA and—unlike the PVB and PVBM series—appeared to be independent of the mol% fraction of VP (Figure 5). No considerable change in water absorption was observed when the VP mol% fraction was reduced from 50–40%, whilst the IBMA mol% fraction was unchanged at 20% (PVBI 50/30/20 cf., PVBI 40/40/20). However, when the VP mol% fraction was kept at 50%, and the IBMA mol% fraction increased from 10 to 15%, water absorption decreased from 2.4% ± 0.6 *w*/*w* to 1.8% ± 0.5 *w*/*w* (52 d) (PVBI 50/40/10 cf., PVBI 50/35/15) (Supplementary Materials, Figure S6). The water absorption for PVBI 50/45/5 appeared to maintain an increasing trend (>~5% *w*/*w* after 40 d) (Figure 5), which was attributed to the low *T_g_* value of the copolymer (*T_g_* = 40.0 °C). The resultant increase in chain mobility likely enabled swelling of the copolymer disc in the presence of water, leading to increased water absorption over time. Importantly, these data (Supplementary Materials, Figure S6) demonstrate that PVBI copolymers could be prepared with a relatively high mol% fraction of VP (50%), and their water absorption properties tuned via the incorporation of IBMA. 

### 3.5. Fluidised-Bed Spray Coating

A model drug formulation and reverse enteric copolymer coatings were applied to Suglets^®^ sugar spheres, to evaluate the performance of the copolymers as taste-masking formulations (Table 2). Initially, Suglets^®^ (dia._avg_ = 1.52 ± 0.08 mm, m_avg_ = 3.36 mg, *n* = 1800) were coated with a PEG/RhB model drug formulation (6:1 PEG:RhB mass ratio). A single coating cycle afforded a 6.2% *w*/*w* mass gain (equiv. to ~209 µg/Suglet^®^), which applied enough of the model drug, RhB (~31 µg/Suglet^®^) to enable inline monitoring of release via UV-vis spectrophotometry. RhB was incorporated as the model drug in the formulation to enable the facile determination of its release, and therefore, the effectiveness of the reverse enteric copolymer coatings applied thereafter. Four sub-batches of Suglets^®^ coated with the model drug formulation (*n* = ~340) (Supplementary Materials, Figure S7) were then each coated with a 10% *w*/*v* reverse enteric copolymer formulation. Each sub-batch was coated using two coating cycles, to afford total mass gains of ~4.9–6.5% *w*/*w*, which were equivalent to ~175–232 µg/Suglet^®^. The same formulation and coating parameters were used for each copolymer formulation, to enable the comparison of their relative performance. Investigation of the effectiveness of the PVBI copolymer formulations, was not conducted due to their poor solubility at pH 2. However, the application of PVBI 50/30/20 is described elsewhere [42]. Generally, Suglets^®^ coated with the PVB or PVBM copolymer formulations did not exhibit any significant signs (qualitatively) of ineffective coating (Supplementary Materials, Figure S8). Twinning was observed between Suglets^®^ following coating with all the formulations, however, it was most pronounced with the PVBM 30/50/20 formulation (Supplementary Materials, Figure S8). However, it is highly likely that this could be mitigated in the future, by further optimisation of the coating parameters.

### 3.6. In Vitro Evaluation of the Copolymers as Reverse Enteric, Taste-Masking Coatings 

To investigate whether the synthesised copolymers could be suitable for use as taste-masking coatings, Suglets^®^ (*n* = 6) bearing each copolymer coating were immersed in a pH 6.8 solution at 37 °C to mimic the human salivary environment. The coatings were considered to have effectively taste-masked the RhB formulation until RhB was observed in the receiving solution. The least effective copolymer formulation was PVBM 30/50/20, where all the tested Suglets^®^ (*n* = 6) released RhB immediately following immersion in the pH 6.8 solution. Moderate taste-masking was observed for the PVB 60/40 copolymer formulation, as sporadic failures for 3/6 Suglets^®^ were observed for up to 30 min, with the remaining Suglets^®^ observed to effectively taste-mask within the same period. In contrast, the most effective copolymer formulations were PVB 40/60 and PVBM 40/50/10, which both effectively contained RhB in all 6 Suglets^®^ for 30 min. Considering the short period of taste-masking required (<5 min) for most oral formulations [7,13], PVB 40/60 and PVBM 40/50/10 showed excellent taste-masking properties. Additional monitoring revealed continued containment of RhB for 72 h, in 5/6 and 6/6 Suglets^®^ for PVB 40/60 and PVBM 40/50/10 coatings, respectively. This result further demonstrated the excellent taste-masking properties of the copolymers and their potential suitability for applications in which taste-masking is required for extended periods (e.g., a liquid oral dosage form [43]). It should be noted that extensive optimisation of the coating parameters and the coated mass of each copolymer was not undertaken, but may afford increases in performance, such as mitigating the single observed failure of a PVB 40/60 Suglet^®^. In conjunction with the low mass gain required to mediate such prolonged taste-masking (cf., 40% or 80% *w*/*w* as reported by Chivate et al.) [13], PVB 40/60 and PVBM 40/50/10 exhibited potential for use as taste-masking reverse enteric coatings with improved performance. 

The dissolution of the copolymer coatings in a simulated gastric environment (pH 1.5 solution, 37 °C) was investigated to determine whether taste-masked formulations would rapidly release their payload following ingestion. The presence of RhB in the receiving solution was monitored inline via UV-vis spectrophotometry (λ_max_ = 556 nm), to determine the initial and complete release times. The results of the release experiments are summarised in Table 3 and release profiles are provided in the Supplementary Materials, Figure S9. In all cases, the release of RhB occurred within 5 min, and was completed within 10 min. As expected, due to their poor taste-masking at pH 6.8, Suglets^®^ coated with PVBM 30/50/20 did not exhibit delayed release at pH 1.5 (i.e., released RhB immediately), and so were not monitored. Both the initial and complete release times for Suglets^®^ coated with PVB 60/40, 40/60, and PVBM 40/50/10, were positively correlated with their taste-masking properties at pH 6.8. For example, only 50% of the tested Suglets^®^ with a PVB 60/40 coating were taste-masked for 30 min, which also exhibited the shortest initial (1.3 ± 0.1 min) and complete release times (2.1 ± 0.3 min). In contrast, all the tested PVBM 40/50/10 coated Suglets^®^ were effectively taste-masked at pH 6.8 and exhibited the longest initial (4.6 ± 1.4 min) and complete release (8.5 ± 2.0 min) times at pH 1.5. 

This behaviour was likely due to the *T_g_* values of the copolymers. PVB 40/60 and PVBM 40/50/10 exhibited similar *T_g_* values (43.3 °C and 45.3 °C, respectively), which were lower than that of PVB 60/40 (58.9 °C). The comparatively lower *T_g_* values of PVB 40/60 and PVBM 40/50/10, likely facilitated the interpenetration of polymer chains [19] during spray coating— due to greater chain mobility—resulting in more effective (i.e., cohesive) and uniform film formation when coated at ambient temperature (23 °C). Coatings that were more cohesive would invariably withstand the artificial salivary environment—which could induce coating-disrupting processes such as water absorption and swelling—better than less cohesive coatings. Similarly, in the gastric environment, cohesive coatings would effectively delay the release of an encapsulated API, due to the necessary dissolution of the coating prior to release. The higher *T_g_* value of PVB 60/40 likely reduced the effectiveness of film formation, leading to thinner and less cohesive coatings that were rapidly disrupted at pH 6.8, and sufficiently solubilised at pH 1.5. The same effect is likely to have occurred for the PVBM 30/50/20 coated Suglets^®^, due to its moderately high *T_g_* value (*T_g_* = 50.2 °C). Suglets^®^ coated with PVB 40/60 or PVBM 40/50/10, were shown to mediate the rapid release of RhB within the simulated gastric environment. Whilst further investigation is required to determine if this performance is conserved at scale, these preliminary experiments suggested that the PVB 40/60 and PVBM 40/50/10 coatings demonstrated an excellent capacity for taste-masking.

## 4. Conclusions

A series of pH-responsive copolymers were synthesised via conventional free radical polymerisation, to produce copolymers suitable for taste-masking. VP constituted the pH-responsive moiety, and was copolymerised with BMA, BMA and MMA, or BMA and IBMA, in varying mol% fractions, to investigate the effects on key physicochemical properties (*T_g_* value, solubility, and water absorption) that affect the taste-masking properties of the resultant copolymers. For the PVB series, the *T_g_* values, solubility and water absorption were all found to be positively correlated with the mol% fraction of VP. In contrast, in the PVBM series, the *T_g_* values were positively correlated with the mol% fraction of MMA, whereas the solubility and water absorption properties were dependent on the mol% fraction of VP. In contrast to the PVB and PVBM series, the solubility, water absorption and *T_g_* values of the PVBI copolymers were all found to be dependent on the mol% fraction of IBMA. Therefore, these properties could be tuned by fixing the mol% fraction of VP and altering the mol% fraction IBMA. Copolymers from the PVB and PVBM series were evaluated preliminarily for their taste-masking effectiveness, via fluidised-bed spray coating. Select copolymers were spray coated onto Suglets^®^ bearing a model drug formulation containing RhB. PVB 40/60 and PVBM 40/50/10 copolymers exhibited excellent taste-masking properties, as they effectively contained RhB for up to 72 h in a simulated salivary environment (pH 6.8). In addition, Suglets^®^ bearing either coating were found to rapidly release their RhB payload within 10 min in a simulated gastric environment (pH 1.5). In both cases, the favourable taste-masking properties were achieved with low mass gain (5.2–6.5% *w*/*w*). Whilst these results are preliminary—given the small batch sizes employed—with further evaluation the reported copolymers may enable the preparation of pharmaceutical formulations with improved taste-masking properties, using relatively low proportions of the copolymers in the resultant dosage form.

## Figures and Tables

**Table 1:** Summary of copolymer repeat unit ratios, molecular weight characteristics, and thermal properties.

Copolymer Code	Targeted mol% of RU	Calculated mol% of RU ^a^	*M_w_*^b^ (kDa)	*M_n_*^b^ (kDa)	*Đ* ^b^	*T_g_*^c^ (°C)
PVB 60/40	60:40	63:37	35.3	21.0	1.7	58.9
PVB 50/50	50:50	51:49	37.1	21.1	1.8	53.1
PVB 40/60	40:60	41:59	30.1	17.5	1.7	43.3
PVBM 40/50/10	40:50:10	40:50:10	32.4	14.8	2.2	45.3
PVBM 30/50/20	30:50:20	30:50:20	34.5	16.2	2.1	50.2
PVBM 30/40/30	30:40:30	31:39:30	35.4	17.2	2.1	59.7
PVBM 40/40/20	40:40:20	41:39:20	36.0	18.6	1.9	57.1
PVBI 50/45/5	50:45:5	49:46:5	25.2	13.2	1.9	40.0
PVBI 50/40/10	50:40:10	50:40:10	25.0	12.1	2.1	52.6
PVBI 50/35/15	50:35:15	51:34:16	27.2	14.2	1.9	64.1
PVBI 50/30/20	50:30:20	51:30:20	34.2	16.4	2.1	73.7
PVBI 40/40/20	40:40:20	40:40:20	43.4	22.3	2.0	70.2

^a^ Monomer conversion and mole ratios determined by GC relative to t_0_ using 1,4-dioxane as an internal reference. ^b^ Determined by GPC using a conventional column calibration of narrow molecular weight PMMA standards. ^c^ Determined by MDSC.

**Table 2:** Summary of Suglet^®^ batch properties before and after coating with model drug and copolymer formulations.

Coating	n_Suglets_^®^	Mass Gain (% *w*/*w*)	Mass Coated (µg/Suglet^®^)
PEG/RhB	1805	6.2	209
PVB 60/40	335	5.2	186
PVB 40/60	332	5.4	193
PVBM 40/50/10	340	6.5	232
PVBM 30/50/20	338	4.9	175

**Table 3:** Summary of in vitro release results of model drug and copolymer coated Suglets^®^.

Coating	Initial Release ^a^ (min)	Complete Release ^b^ (min)
PVB 60/40	1.3 ± 0.1 (10)	2.1 ± 0.3 (15)
PVB 40/60	3.6 ± 1.5 (43)	7.2 ± 2.4 (33)
PVBM 40/50/10	4.6 ± 1.4 (32)	8.5 ± 2.0 (23)
PVBM 30/50/20	-	-

^a^ Initial release was defined as 5% of the total release of RhB. ^b^ Complete release was defined as 90% of the total release of RhB. All values are reported as mean ± std. dev. (%RSD) for *n* = 5 samples.

## Data Availability

The data presented in this study are available on request from the corresponding author.

## Supplementary Materials

The following supporting information can be downloaded at: https://www.mdpi.com/article/10.3390/pharmaceutics15020454/s1, Figure S1: Normalised differential reactive index GPC chromatograms of the copolymer series; Figure S2: Scheme showing synthesis of PVB copolymers and ^1^H NMR spectra of the PVB series of copolymers; Figure S3: Scheme showing synthesis of PVBM copolymers and ^1^H NMR spectra of the PVBM series of copolymers; Figure S4: Scheme showing synthesis of PVBI copolymers and ^1^H NMR spectra of the PVBI series of copolymers; Figure S5: GPC calibration curves prepared for the copolymer series; Table S1: Summary of copolymer solubilities as determined via GPC; Figure S6: Summary of water absorption (% *w*/*w*) results for the copolymer series; Figure S7: Suglets^®^ coated with the model drug formulation containing PEG and RhB; Figure S8: Suglets^®^ coated with PVB 60/40, PVB 40/60, PVBM 40/50/10, and PVBM 30/50/20 copolymer formulations; Figure S9: Release profiles obtained of Suglets^®^ coated with a PEG/RhB model drug formulation and PVB 60/40, PVB 40/60, and PVBM 40/50/10, during dissolution in a pH 1.5 solution.
