# Supplementary material for: Composition-Property Relationships of pH-Responsive Poly[(2-vinylpyridine)-co-(butyl methacrylate)] Copolymers for Reverse Enteric Coatings
Source: Pharmaceutics. 2023 Jan 30;15(2):454. doi: 10.3390/pharmaceutics15020454 (PMC9959453; doi:10.3390/pharmaceutics15020454)
Supplement: Supplementary file 1 [file pharmaceutics-15-00454-s001.zip › pharmaceutics-2103306-supplementary.pdf]

pharmaceutics

MDPI

Supplementary Materials: Composition-Property Relationships of pH-Responsive Poly[(2-vinylpyridine)-co-(butyl methacrylate)] Copolymers for Reverse Enteric Coatings

Kyle Brewer and Anton Blencowe

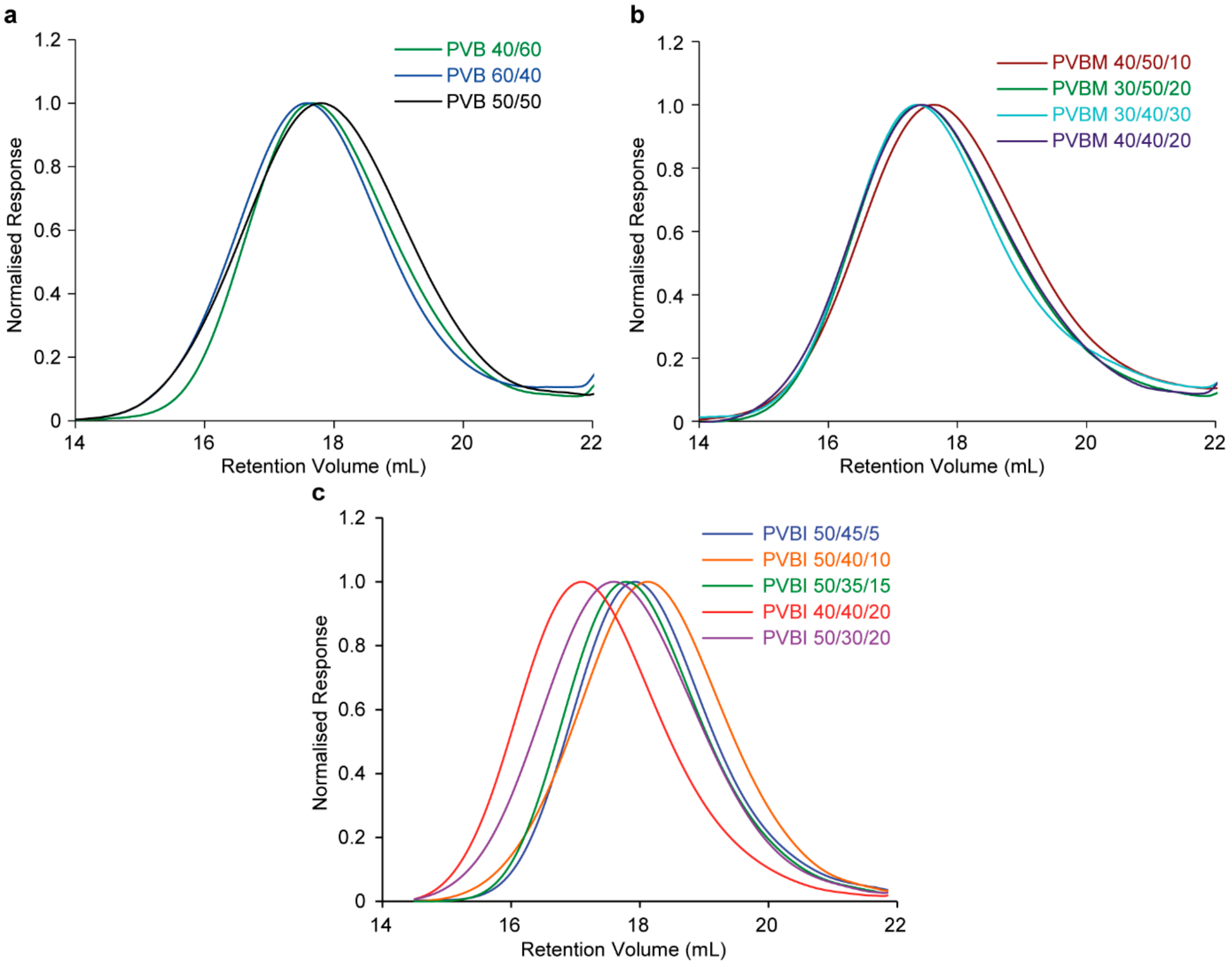

Figure S1. Normalised differential reactive index GPC chromatograms of the copolymer series.

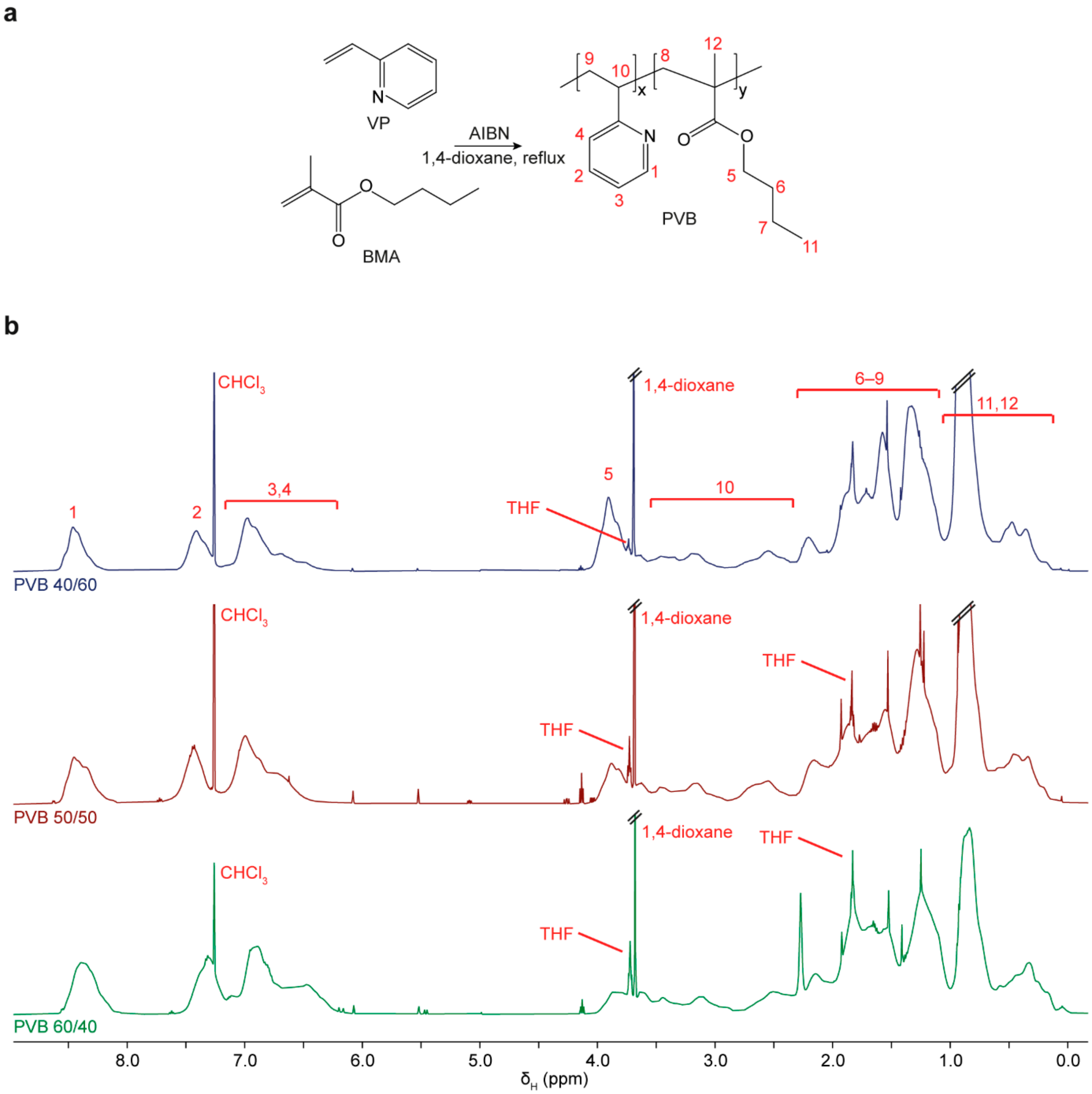

Figure S2. a) Scheme showing synthesis of PVB copolymers and b) ^{1}H NMR spectra (600 MHz, 25 °C, $CDCl_3$) of the PVB series of copolymers.

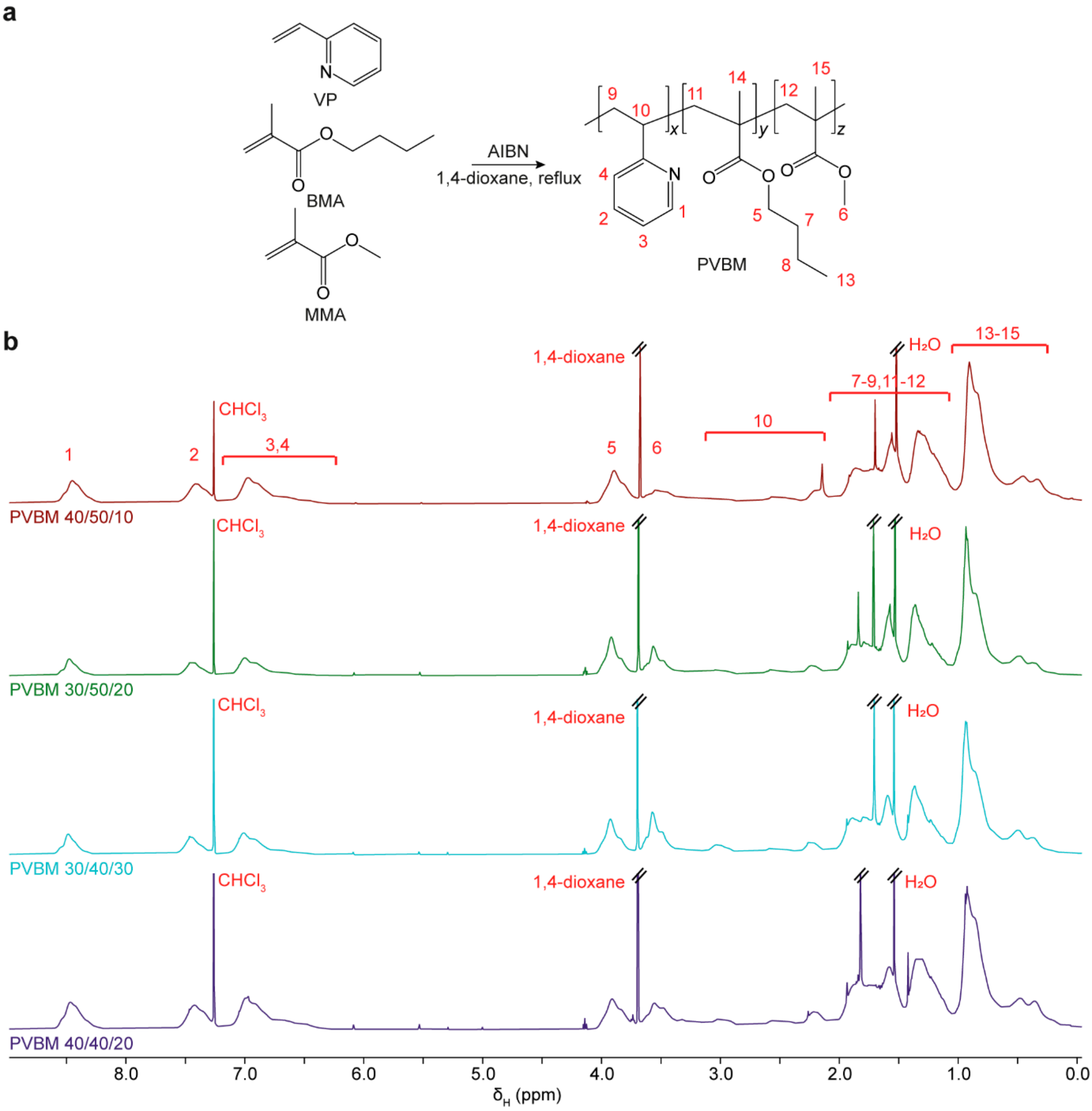

Figure S3. a) Scheme showing synthesis of PVBM copolymers and b) ^{1}H NMR spectra (600 MHz, 25 °C, $CDCl_3$) of the PVBM series of copolymers.

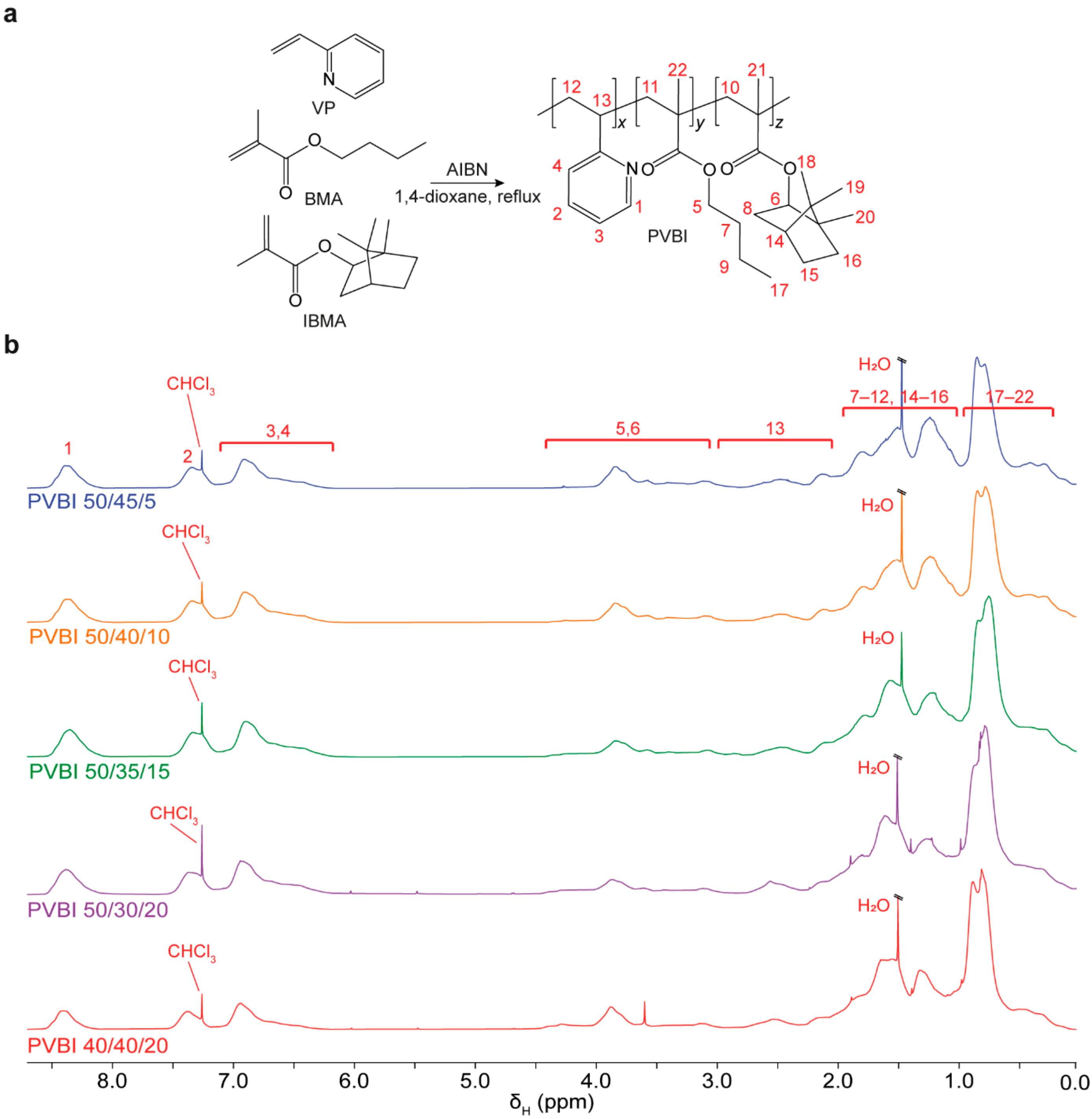

Figure S4. a) Scheme showing synthesis of PVBI copolymers and b) ^{1}H NMR spectra (600 MHz, 25 °C, $CDCl_3$) of the PVBI series of copolymers.

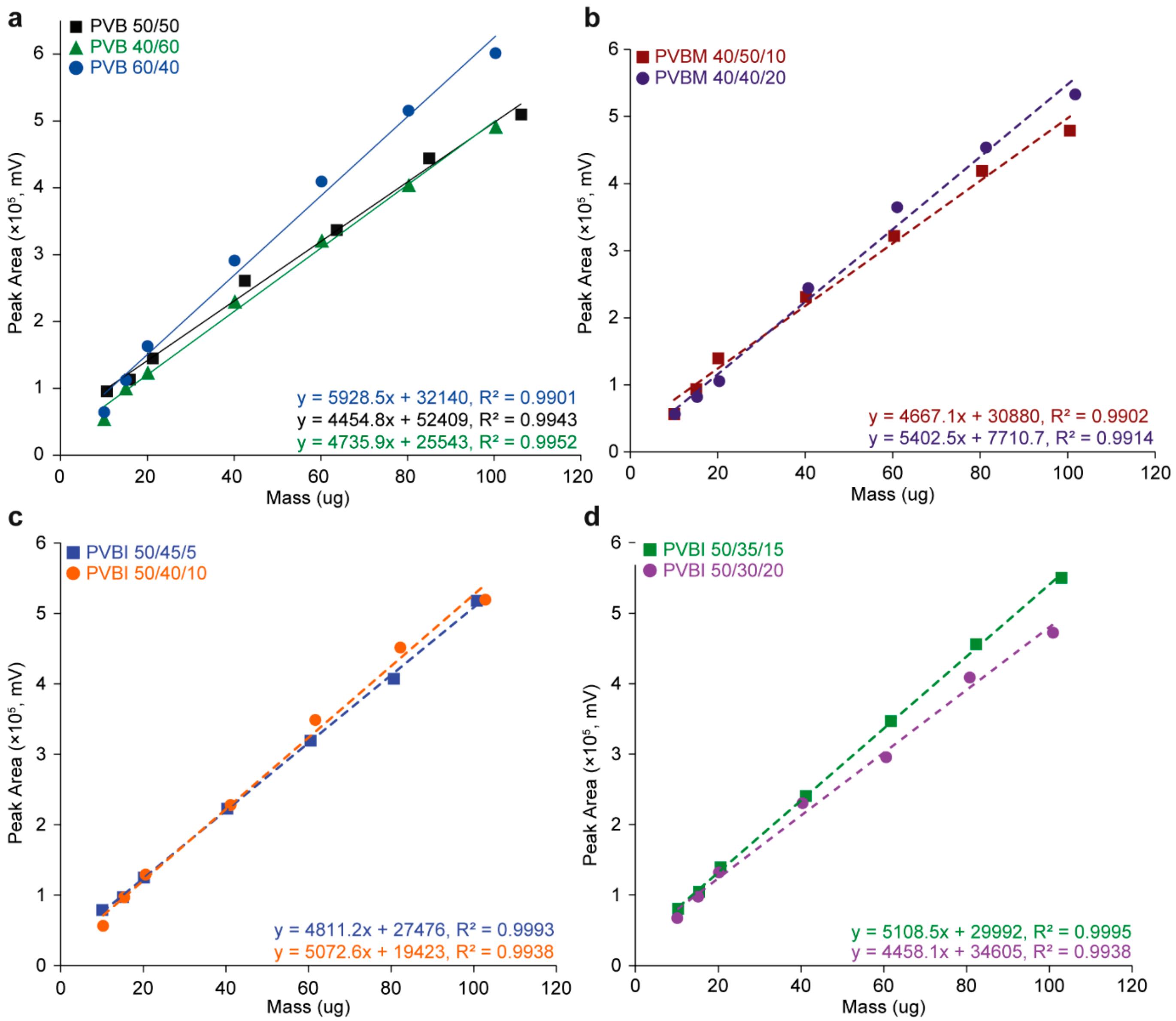

Figure S5. GPC calibration curves prepared for the copolymer series, from aqueous solutions of each copolymer (10 mg.mL^{-1}, pH 1.0), injected at volumes between 10–100 µL.

Table S1. Summary of copolymer solubilities as determined via GPC.

Copolymer Code	Solubility (mg.mL^{-1})[a]		
	**pH 1.0**	**pH 1.5**	**pH 2.0**
PVB 50/50	7.2 ± 0.5	7.5 ± 1.4	2.3 ± 0.5
PVB 60/40	10.9 ± 1.0	9.8 ± 1.2	4.3 ± 0.3
PVB 40/60	8.6 ± 0.5	7.1 ± 0.3	3.5 ± 0.2
PVBM 40/50/10	6.5 ± 0.2	5.4 ± 0.6	2.3 ± 0.1
PVBM 40/40/20	6.1 ± 0.4	5.6 ± 0.3	2.1 ± 0.1
PVBI 50/45/5	10.4 ± 0.2	6.3 ± 0.1	1.4 ± 0.1
PVBI 50/40/10	9.9 ± 0.7	5.7 ± 0.2	-
PVBI 50/35/15	9.5 ± 0.7	4.9 ± 0.2	-
PVBI 50/30/20	10.5 ± 0.3	3.7 ± 0.4	-

[a] Copolymer solubility reported as mean ± std. dev. (n = 3).

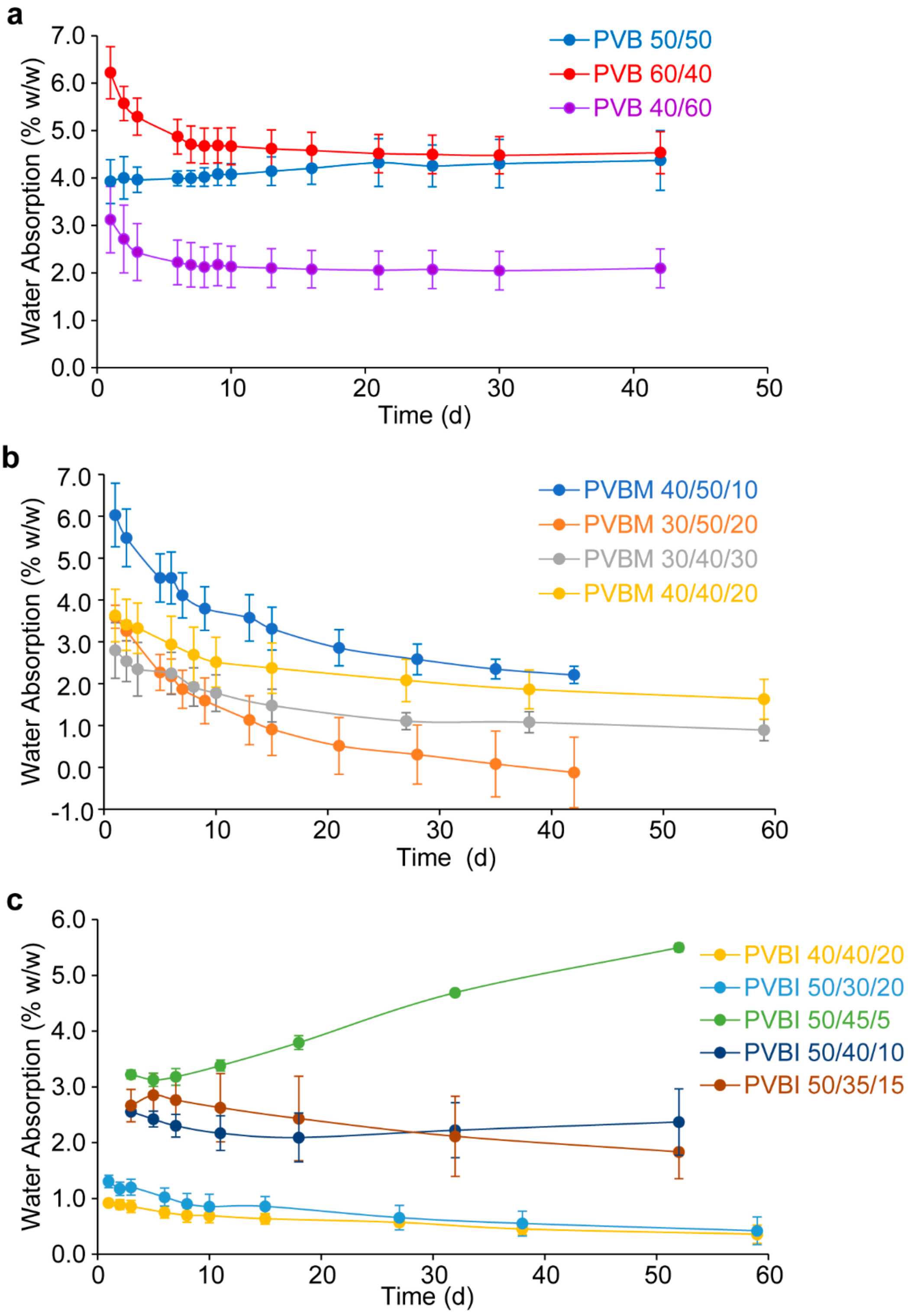

Figure S6. Summary of water absorption (% w/w) results for the copolymer series, following ~ 40–60 d immersion in PBS (pH 7.4, 37 °C). Values are reported as mean ± std. dev. (n = 3).

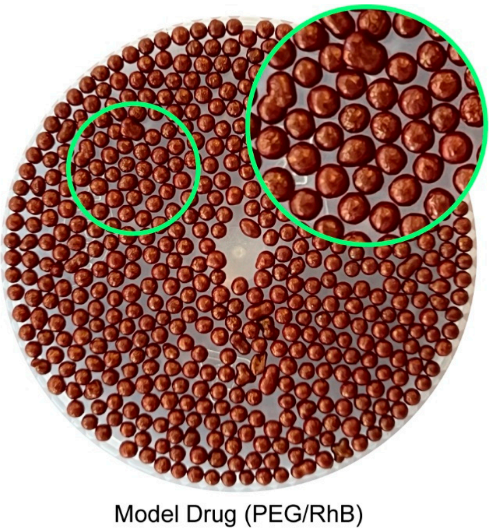

Figure S7. Suglets® coated with the model drug formulation containing PEG and RhB, coated at a mass gain of 6.2% w/w.

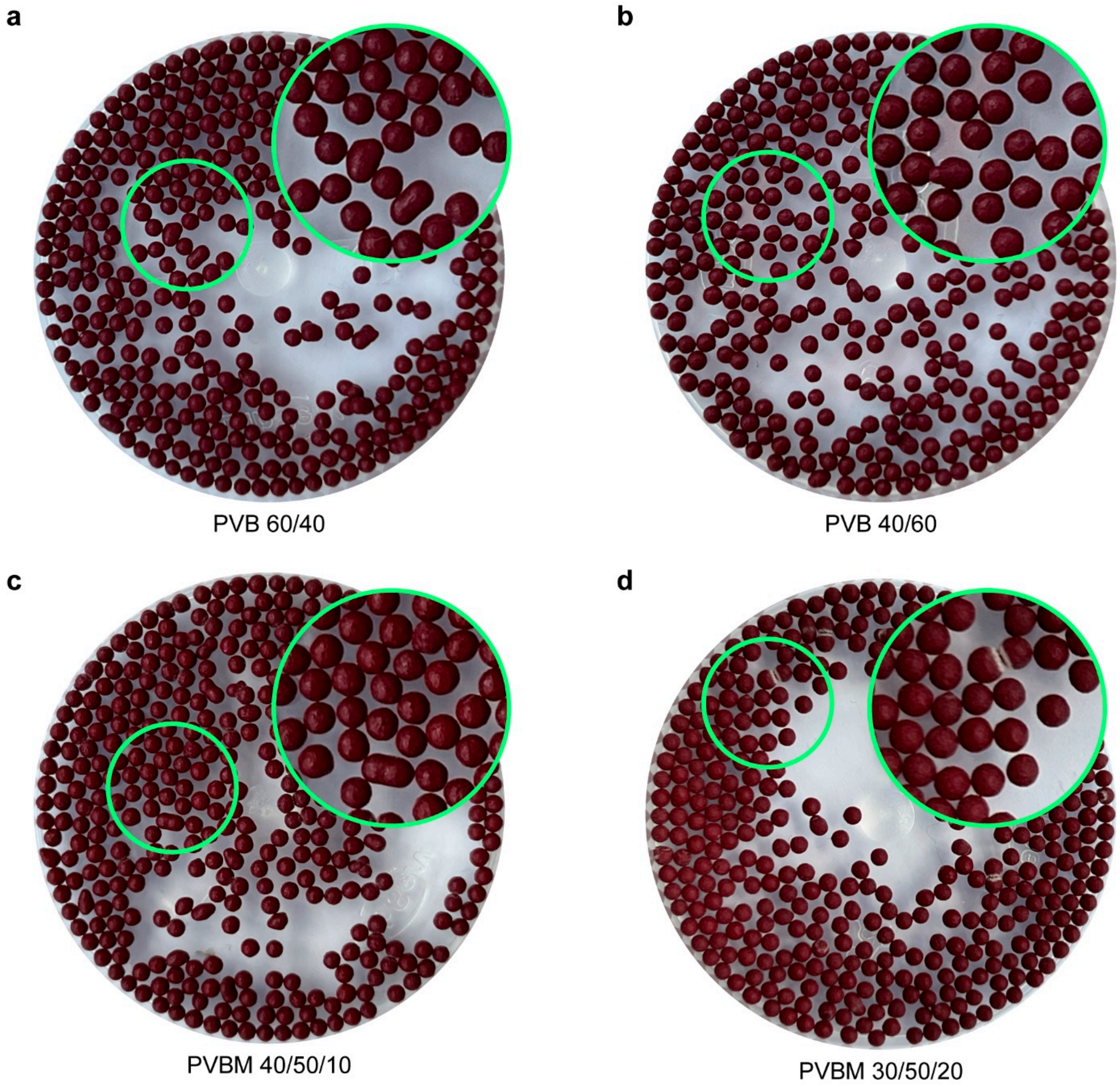

Figure S8. Suglets® coated with a) PVB 60/40 (5.2% w/w mass gain), b) PVB 40/60 (5.4% w/w mass gain), c) PVBM 40/50/10 (6.5% w/w mass gain), and d) PVBM 30/50/20 (4.9% w/w mass gain), co-polymer formulations.

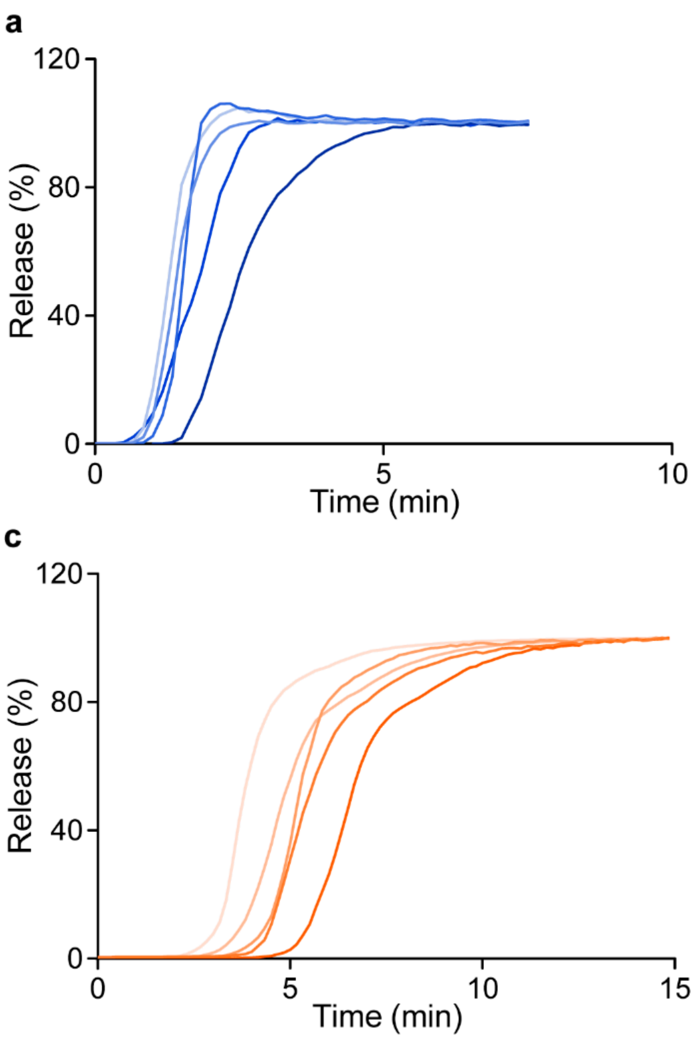

Figure S9. Release profiles obtained of Suglets® coated with a PEG/RhB model drug formulation (6.2% w/w mass gain), and a) PVB 60/40 (5.2% w/w mass gain), b) PVB 40/60 (5.4% w/w mass gain), and c) PVBM 40/50/10 (6.5% w/w mass gain), during dissolution in a pH 1.5 solution (37 °C), under stirring. Traces represent individual samples (n = 5), monitored via the absorbance maximum of rhodamine b (λ_{max} = 556 nm). Note that in a) two samples show a > 100% release peak maximum, which was caused by a local increase in dye concentration, due to a lag in stirring during analysis.
